# Should we adopt a prognosis-based approach to unexplained infertility?

**DOI:** 10.1093/hropen/hoac046

**Published:** 2022-10-13

**Authors:** Laxmi Shingshetty, Abha Maheshwari, David J McLernon, Siladitya Bhattacharya

**Affiliations:** Aberdeen Centre for Reproductive Medicine, NHS Grampian, Aberdeen, UK; Aberdeen Centre for Reproductive Medicine, NHS Grampian, Aberdeen, UK; Medical Statistics Team, School of Medicine, Medical Sciences and Nutrition, University of Aberdeen, Aberdeen, UK

**Keywords:** unexplained infertility, prediction models, expectant management, treatment, assisted conception, spontaneous pregnancy, live birth

## Abstract

The treatment of unexplained infertility is a contentious topic that continues to attract a great deal of interest amongst clinicians, patients and policy makers. The inability to identify an underlying pathology makes it difficult to devise effective treatments for this condition. Couples with unexplained infertility can conceive on their own and any proposed intervention needs to offer a better chance of having a baby. Over the years, several prognostic and prediction models based on routinely collected clinical data have been developed, but these are not widely used by clinicians and patients. In this opinion paper, we propose a prognosis-based approach such that a decision to access treatment is based on the estimated chances of natural and treatment-related conception, which, in the same couple, can change over time. This approach avoids treating all couples as a homogeneous group and minimizes unnecessary treatment whilst ensuring access to those who need it early.

## Introduction

Infertility, defined as an inability to establish a clinical pregnancy after 12 months of regular, unprotected sexual intercourse (Zegers-Hochschild *et al*., 2017), affects one in six couples (Oakley *et al.*, 2008). Although known causes include anovulation, tubal pathology, endometriosis and abnormal semen parameters (Bostofte *et al.*, 1993), standard tests fail to reveal any abnormalities in a quarter of all infertile couples (Brandes *et al.*, 2011; Ray *et al.*, 2012) whose infertility is described as unexplained. The term ‘unexplained’ itself is contestable, as it probably reflects the inadequacy of standard fertility investigations in successfully identifying any underlying causes which might contribute to a potentially complex condition (Gleicher and Barad, 2006).

In the absence of an identifiable barrier to conception, natural conception remains a possibility (Brandes *et al.*, 2011; van Geloven *et al.*, 2013) for couples with unexplained infertility, but many are sceptical about an expectant approach and are keen to receive treatment. Planning specific treatment is challenging in the absence of a clear underlying pathology, but clinicians have historically attempted to treat unexplained infertility by boosting ovulation and/or partially bypassing some of the physiological steps involved in reproduction.

Clomiphene citrate, an effective treatment for anovulation, has been used commonly in the past in an attempt to stimulate multifollicular ovulation, although its antiestrogenic property can decrease endometrial thickness and compromise implantation. Inexpensive and available as an oral preparation, it is still prescribed as the sole treatment in certain settings, despite data from randomized controlled trials demonstrating its ineffectiveness in this context (Hughes *et al.*, 2010). IUI and IVF are commonly used treatments in couples with unexplained infertility (Mol *et al.*, 2018). IUI overcomes a potential cervical barrier by depositing washed sperm within the uterus, while IVF bypasses *in vivo* processes within the female genital tract, including fertilization. Given the ever-present chance of natural conception in these patients, the inability to offer each couple an individualized prognosis (Jing *et al.*, 2021) makes it difficult to agree on a management plan which incorporates a transition from an expectant phase to active fertility treatment.

## Current approach to treatment and outcomes

Although recent advances in reproductive medicine have not been successful in resolving our understanding of unexplained infertility, greater access to assisted reproduction over the past few decades has resulted in an exponential growth in the number of couples presenting for treatment (Ferraretti *et al.*, 2017). While invasiveness, acceptability and costs are key factors in clinical decision-making (Pandian *et al*., 2003), the couple’s preference for any form of treatment over expectant management is driven by a firm belief in the effectiveness of treatment, along with a general lack of awareness of the likelihood of natural conception (ESHRE Capri Workshop Group, 2017).

Despite being aware of the value of expectant management in couples with a short duration of infertility, this approach has never been popular (Kersten *et al.*, 2015) with clinicians who have tended to favour a treatment pathway based on escalating degrees of invasiveness and/or expense. IUI, which is less invasive and costs less per cycle in comparison with IVF (Brandes *et al.*, 2011; Buckett and Sierra, 2019), is often seen as the first step. This is generally accompanied by ovarian stimulation IUI (OS IUI), which has been shown to increase its effectiveness (Zolton *et al.*, 2020) although it can potentially increase the risk of a multiple pregnancy (Buckett and Sierra, 2019).

While it does not necessarily guarantee success (Moragianni and Penzias, 2010), IVF is, increasingly, an attractive option for many couples as it can provide a quicker route to pregnancy (Ray *et al.*, 2012). A Cochrane review has shown it to be more effective than unstimulated IUI and demonstrated its superiority over OS IUI in couples who have previously received IUI, but not those who are treatment naïve (Pandian *et al.*, 2012). The additional invasiveness of ICSI has not been shown to confer any additional benefit over conventional IVF (Dang *et al.*, 2021) in unexplained infertility.

## Guidance: national guidelines and recommendations for professional societies

The American Society for Reproductive Medicine (Practice Committee of the American Society for Reproductive Medicine, 2020) does not recommend Clomiphene on its own but suggests a policy of OS IUI (initially with oral and then with parenteral agents), followed by IVF. In the UK, current National Institute for Health Care and Excellence (NICE) guidance does not support funding for OS IUI and recommends IVF (ideally with elective single embryo transfer) in couples with 2 years of unexplained infertility (NICE, 2017). This decision is driven by the need to offer a definitive treatment, which is most likely to be successful, rather than a phased approach involving first OS IUI, followed by IVF. It also reflects concern about a potentially higher risk of iatrogenic multiples associated with ovarian stimulation in ovulatory women as opposed to IVF using an elective single embryo transfer strategy. Neither guideline acknowledges the need to incorporate the chance of natural conception in individual couples in coming to a decision regarding when to initiate active treatment. Although both guidelines acknowledge the impact of female age on fertility, neither takes this and other prognostic factors into consideration whilst planning treatment and therefore risk overtreating some couples, whilst causing unacceptable delay in others (NICE, 2017).

The Dutch fertility guideline (www.nvog.nl) recommends the use of a prognostic model (www.freya.nl) to inform clinical decision-making around initiation of active treatment (Hunault *et al.*, 2004). Clinicians are advised to recommend a 6- to 12-month period of expectant management for couples deemed to have a good prognosis (>30% chance of conception over the next 12 months), but compliance is not universal and 36% of Dutch couples have been shown to be overtreated (Kersten *et al.*, 2016). Elsewhere, clinicians do not use a prognostic model to make key decisions around the timing and nature of treatment, although evidence from a number of randomized trials is supportive of this approach (Chua *et al.*, 2020; Wang *et al.*, 2020). For example, OS IUI is not better than expectant management in couples with an intermediate prognosis (30–40% chance of conception over the next 12 months) but very effective in couples with a poor prognosis (<30% probability) (Farquhar *et al*., 2018). In women in their late 30s, immediate IVF leads to higher success rates in comparison with OS IUI (Goldman *et al.*, 2014). Current unexplained infertility guidelines also do not offer any advice for couples who have undergone unsuccessful IVF treatment, although some of them will conceive naturally over time (ElMokhallalati *et al.*, 2019).

Although clinicians and couples make intuitive decisions about expediting treatment in older women and in those with prolonged infertility, current treatment strategies do not formally estimate the chances of conception (with and without active treatment) in a couple or favour a prognosis-based plan on when, and how, to treat them (Ray *et al.*, 2012). Without knowledge of the added value of active treatment (over and above the background chances of natural conception) for each couple, as well as the associated risks and costs, it is difficult to be confident that couples and clinicians are genuinely able to make an informed decision.

## A prognosis-based approach

Prognostic models based on real-life clinical data offer a way of individualizing treatment in couples with unexplained infertility in order to optimize the chances of success, whilst minimizing exposure to unnecessary, expensive and invasive interventions. Such models could facilitate decision-making around timing and choice of treatment. This approach is not new to healthcare; a predictive model called ‘PREDICT’ is a prognostic and treatment benefit model implemented online to estimate the chances of survival after the early stage of breast cancer (Candido Dos Reis *et al.*, 2017; Webb *et al.*, 2022). Other prognostic models have been used to individualize care across different healthcare settings including suicide prevention, cardiovascular risk assessment and opioid-related adverse events (Claassen *et al.*, 2014; Damen *et al.*, 2016). The Framingham risk score, a gender-specific algorithm to estimate 10-year cardiovascular risk, is a simple and efficient prediction tool which is widely used in primary care to stratify care (D’Agostino *et al*., 2008). The increasing use of electronic health records is providing more opportunities for data-driven models to inform decision-making in clinical settings (Evans, 2016).

Prognostic models determine the natural course of a condition, whereas predictive models estimate the chances of responding successfully to treatment. Both are needed to underpin a prognosis-based approach, which can reduce the need for unnecessary intervention, expedite access to necessary treatment and manage couples’ expectations (Kersten *et al.*, 2016). This approach can also reverse the current trend towards over-medicalization of reproduction (Kamphuis *et al.*, 2014) thus reducing treatment-related risks such as ovarian hyperstimulation syndrome, multiple pregnancies and preterm birth (Pandey *et al.*, 2012; Luke *et al.*, 2017).

A key prognostic factor in unexplained infertility is female age, which has a major impact on the chances of conception both with and without treatment. Unexplained infertility is also more commonly reported in women who are older—accounting for four out of five women over 40, compared to 1 in 10 in women under 35 years of age (Broer *et al.*, 2011; Somigliana *et al.*, 2016)—although it is important to avoid conflating age-related infertility with unexplained infertility (ESHRE Capri Workshop Group, 2017). While couples with unexplained infertility have a genuine chance of natural conception (Brandes *et al.*, 2011), time has a critical impact on prognosis acting through both female age and duration of infertility (ESHRE Capri Workshop Group, 2017).

A prognosis-based approach could ensure that women who are young and have a better prognosis could benefit from a longer period of expectant management while those who are older, with a poorer chance of natural conception, could have early treatment (Bhattacharya *et al.*, 2021). Conversely (ESHRE Capri Workshop Group, 2017), it might be reasonable to offer early treatment to young women if their chance of natural conception is low (e.g. <30% over 12 months) and further delay is likely to affect the chances of treatment-related success (Bensdorp *et al.*, 2017).

In addition to their utility in planning treatment in couples with unexplained infertility, population-based prognostic models, which can estimate chances of natural pregnancy in a general population, could be used to generate a fertility score, not unlike the recently developed Endometriosis Fertility Index (Becker *et al*., 2022). Couples with an excellent fertility score could be encouraged to continue expectant management, reducing the number of unnecessary referrals and invasive therapies. Conversely, those with a low fertility score could be fast tracked for investigations and active treatment without having to wait a full year. In a sense, this is already occurring in clinical practice where intuitive reasoning has led to guidelines supporting early referral to specialist care for women over 35 years of age (National Collaborating Centre for Women’s and Children’s Health (UK), 2013). A prognosis-based approach could lead to health economic benefits by expediting access to timely treatment for those who need it, whilst avoiding overtreatment in others with a good chance of natural pregnancy.

## Prognostic models and their limitations

A prognosis-based paradigm of decision-making is predicated on patients and clinics having access to accurate prognostic and predictive models which can determine the chances of conception with and without treatment. The Hunault model, currently in use in the Netherlands, can only be used once—at the point of completion of fertility investigations—to estimate chances of spontaneous conception over the subsequent 12 months leading to live birth (Hunault *et al.*, 2004). This has limitations in terms of providing revised estimates for pregnancy in couples who return after being advised of a period of expectant management, as the model cannot be used again. A model which can provide repeated estimates of treatment-related and treatment-independent chances of conception at different points in time is critical to a prognosis-based approach in a clinical setting (McLernon *et al.*, 2014, 2019). A number of models have been developed to estimate chances of natural conception in unexplained infertility (Bostofte *et al.*, 1993; Leushuis *et al.*, 2009; McLernon *et al.*, 2014; van Eekelen *et al.*, 2017). Others have attempted to predict the chances of conception associated with IUI (Leushuis *et al.*, 2009; van Eekelen *et al.*, 2019) and IVF (Templeton *et al.*, 1996; Nelson and Lawlor, 2011; Ratna *et al.*, 2020) or both (McLernon *et al.*, 2019).

The key predictors in the Hunault model include female age, duration of infertility, previous pregnancy, referring clinician (general practitioner or gynaecologist), number of motile sperm and the outcome of a post-coital test (Hunault *et al.*, 2004; ESHRE Capri Workshop Group, 2017; Mol *et al.*, 2018). Based on the estimated chance of conception over the next 12 months, clinicians can identify couples with a >40% chance of conception who can be managed expectantly while those whose chances of conceiving are <20% will begin active treatment straight away. In couples with an intermediate prognosis (20–40%), other factors, such as patients’ expectations and duration of infertility, can be considered. Although it allows a personalised approach to decision-making and potentially avoids unnecessary intervention (Kersten *et al.*, 2016; Jing *et al.*, 2021), its major drawback is that it cannot be used to reassess a couple’s chances of conception at different points in time.

This limitation can be overcome by dynamic prediction models, which are able to estimate changes in prognosis in any individual couple over time (ESHRE Capri Workshop Group, 2017). A dynamic model devised by van Eekelen *et al.* (2017) has used Dutch data to estimate, at different points in time, the chances of natural conception over a 12-month time horizon leading to an ongoing pregnancy. A second dynamic model (McLernon *et al.*, 2019) is able to calculate monthly changes in the chance of natural conception (leading to live birth) over a time horizon of 6 months and compare this with estimated chances of conception through OS IUI and IVF (McLernon *et al.*, 2019). The limitations of this model are that it is based on observational data from a single centre, does not include potentially important predictors, such as BMI and ethnicity, and needs further refinement using larger datasets in order to improve its accuracy.

Existing models have tended to rely on the analysis of relatively small- to moderate-sized datasets using multivariable regression techniques. Given the move towards electronic health records, it can be speculated that the interrogation of high dimensional clinical datasets by means of machine learning approaches, such as Bayesian neural networks and boosting algorithms, could be used in future to make clinical predictions with high levels of accuracy. Through unsupervised and semi-supervised model updating processes, these algorithms have the potential of being able to automatically rectify any calibration drift related to changes in clinical practice and case mix (Siristatidis *et al.*, 2016; Curchoe *et al.*, 2020). These automated approaches will need regular validation checks to ensure that predictive accuracy is not compromised. Reporting guidelines and risk of bias tools are currently being developed for prediction model studies based on machine learning (Collins *et al.*, 2021).

Machine learning can analyse complex data, identify patterns and generate a predictive algorithm, but the insight and contextual information from clinicians remains critically important (Lee, 2020). Machine learning approaches may also be at risk of bias—especially with models based on smaller datasets (Andaur Navarro *et al.*, 2021)—and all models need to be validated before they can be used in clinical practice (Linardatos *et al.*, 2021; Sarker, 2022). It is pertinent to emphasize that studies have shown no significant benefit of artificial intelligence-based modelling over traditional regression-based modelling, suggesting that the hypothesis of enhanced accuracy from the former is purely speculative (Lynam *et al.*, 2020; Liew *et al.*, 2022).

## Next steps

Although prognostic and prediction models in infertility have been available for a few years, their uptake has been limited, both owing to a lack of confidence in their usefulness and practical challenges in embedding them within clinic protocols (Zhang *et al.*, 2020). Even in the Netherlands, where they are supported by national guidance, their use around the initiation of active treatment is by no means universal (Kersten *et al.*, 2015). For a prognosis-based approach to unexplained infertility to become part of routine clinical practice, several conditions need to be fulfilled. The first is the availability of accurate models customized for local populations, which can provide estimates of conception with and without active treatment in individual couples. This is best achieved by thorough updating and validation of existing models using national databases or information from multiple clinics (Steyerberg and Harrell, 2016). This process can be challenging, given that universal use of electronic health records is still not a reality in many settings and clinics still use paper records without the ability to link diagnostic, treatment and outcome data to each couple. Where this is possible, the incorporation of appropriate models into patient management systems will allow live predictions to be made at the time of consultation. A prediction model embedded within a large electronic health record system will also allow models to be updated at regular intervals and their performance improved over time by correlating predicted with observed outcomes (Su *et al.*, 2018). Until such time as high dimensional electronic health records are commonly available in reproductive medicine, we advise that these models be developed using regression modelling approaches which incorporate baseline (and possibly time-varying) prognostic factors and treatment over time (van Houwelingen and Putter, 2011; Rizopoulos, 2012). Such models need to be developed carefully with appropriate adjustment for prognostic factors, which allow an unconfounded estimate of the effect of treatment using a counterfactual prediction framework (van Geloven *et al.*, 2020). This approach tries to model the actual effect of the treatment for the individual and should be the preferred method for dynamic prediction of the changing effect of treatment over time. This will help clinicians make decisions on when to commence treatment in couples with unexplained infertility. However, the dynamic modelling approach requires a large sample size and availability of important confounders—a limitation of the McLernon *et al.* (2019) model.

Once accurate and user-friendly models are available, clinicians and patients need to be made aware of their potential benefits. Confidence in the estimates provided is crucial, as is their perceived additional value over intuitive decisions, which take into account the costs, risks and benefits associated with treatments (Kappen *et al.*, 2018). A transition to a prognosis-based approach will require a thorough exploration of barriers and facilitators, and an active implementation plan that addresses these at personal and organizational levels within the fertility setting. Ultimately, robust evaluation will be necessary to demonstrate whether this strategy genuinely improves outcomes for couples with unexplained infertility. In order to enhance the uptake and strength of prediction models, there is a timely need for robust evidence in the form of randomized controlled trials to demonstrate the benefit of these models in clinical practice. Certainty in the benefits could prove vital for the keen adoption of these models into routine use. Such an evidence-based approach would impart change in clinical practice and gain confidence amongst the patients.

## Conclusion

Unexplained infertility lends itself naturally to a prognosis-based approach to key decisions around when to treat and what treatment is best. The benefit of any active treatment modality needs to be interpreted in the context of the anticipated chance of natural conception. Current prediction tools have the potential to be used in routine clinical decision-making but need to be further refined for this purpose.

## Data Availability

No new data have been generated or analysed in support of this publication.
